# Correlation of organelle dynamics between light microscopic live imaging and electron microscopic 3D architecture using FIB-SEM

**DOI:** 10.1093/jmicro/dfaa071

**Published:** 2020-11-20

**Authors:** Keisuke Ohta, Shingo Hirashima, Yoshihiro Miyazono, Akinobu Togo, Kei-ichiro Nakamura

**Affiliations:** Advanced Imaging Research Center, Kurume University School of Medicine, 67 Asahi-machi, Kurume, Fukuoka 830-0011, Japan; Department of Anatomy, Kurume University School of Medicine, 67 Asahi-machi, Kurume, Fukuoka 830-0011, Japan; Department of Anatomy, Kurume University School of Medicine, 67 Asahi-machi, Kurume, Fukuoka 830-0011, Japan; Department of Anatomy, Kurume University School of Medicine, 67 Asahi-machi, Kurume, Fukuoka 830-0011, Japan; Advanced Imaging Research Center, Kurume University School of Medicine, 67 Asahi-machi, Kurume, Fukuoka 830-0011, Japan; Department of Anatomy, Kurume University School of Medicine, 67 Asahi-machi, Kurume, Fukuoka 830-0011, Japan

**Keywords:** CLEM, live imaging, focused ion beam, mitochondria, dynamics, fragmentation

## Abstract

Correlative light and electron microscopy (CLEM) methods combined with live imaging can be applied to understand the dynamics of organelles. Although recent advances in cell biology and light microscopy have helped in visualizing the details of organelle activities, observing their ultrastructure or organization of surrounding microenvironments is a challenging task. Therefore, CLEM, which allows us to observe the same area as an optical microscope with an electron microscope, has become a key technique in cell biology. Unfortunately, most CLEM methods have technical drawbacks, and many researchers face difficulties in applying CLEM methods. Here, we propose a live three-dimensional CLEM method, combined with a three-dimensional reconstruction technique using focused ion beam scanning electron microscopy tomography, as a solution to such technical barriers. We review our method, the associated technical limitations and the options considered to perform live CLEM.

## Introduction

**Fig. 1.**
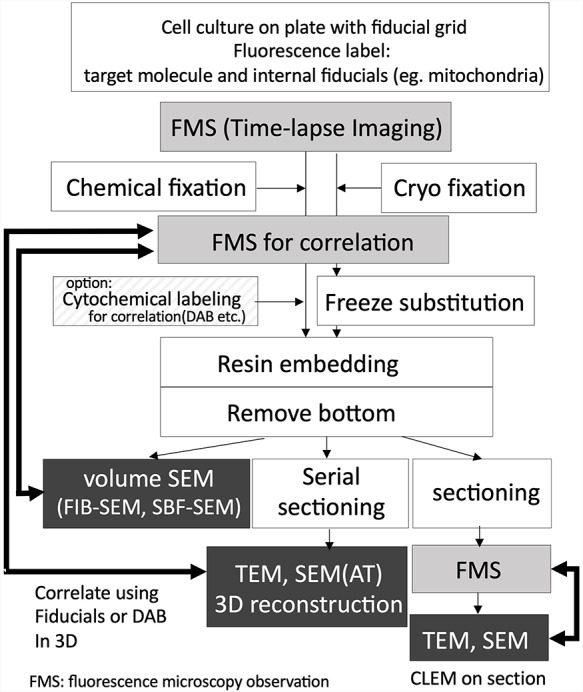
Workflow of the live CLEM combined with light microscopic time-lapse imaging and EM. Fluorescence microscope imaging process using wide-field microscope, confocal microscopy, multiphoton microscopy, and types of super-resolution microscopies. Dark boxed area indicating correlative observation process using EM. Volume SEM, including FIB-SEM and SBF-SEM, which are automated 3D reconstruction methods.

Organelle dynamics is one of the most important topics in the field of cell biology. Interaction or crosstalk between organelles through membrane contact is considered to form networks of functional modules of cellular processes, all of which occur in the microenvironment of the cell at a level of tens of nanometers. Although most of these microenvironmental processes occur in short time spans of <1 s, the structural background of the microenvironment as a reaction site is extremely dynamic and changes within seconds. Such dynamics can be analyzed by live imaging of fluorescently labeled organelles or target molecules using fluorescence microscopy. In addition, the recent advances in super-resolution microscopy have allowed us to obtain a resolution of tens of nanometers and have made it possible to visualize the intracellular events more clearly. However, fluorescence microscopy is limited to visualizing the location of fluorescent probes and does not provide structural and morphological information surrounding the probes. Additionally, significantly high spatial resolution is required to understand membrane–membrane contacts and sub-organellar structural changes. Thus, electron microscopy (EM) is utilized, which is a unique technique that provides morphological information with a higher resolution than modern super-resolution microscopy. However, it is extremely difficult to observe live events of biological phenomena because of their mechanism, allowing us to observe only a snapshot of the events. Correlative light and electron microscopy (CLEM) is a technique that can overcome such technical problems to satisfy these life science needs. The CLEM method uses two microscopic modalities, an optical microscope and EM, which examine the same sample. The dynamics of cellular process or overall structures of specimens are acquired using an optical microscope, and then, the specimens are fixed at a certain point in time. Finally, the EM observations provide a detailed ultrastructure of the area observed by the optical microscope and correlated both images. CLEM is a group of methods, rather than a single method, which achieves collative observation between light and electron microscopies on the same sample. Although CLEM has considerable advantages for biological research, it has many technical issues; for instance, the same samples must be fixed, morphologically preserved and reobserved for both modalities. Therefore, various methods have been developed since the 1960s, depending on the purpose of the observation [1–3]. Various optical microscopes are employed for CLEM, including conventional wide-field light microscopes and recent super-resolution microscopes. In contrast, most early work in EM was performed using transmission electron microscopy (TEM); however, recently, scanning electron microscopy (SEM) has become more commonly used for CLEM procedures. Volume SEM or serial slice SEM methods, which enable the visualization of the three-dimensional (3D) cellular architecture by serial face observation of the specimen, have been frequently employed for CLEM works in recent years (Fig. 1). Depending on the wide range of options and setup, various methods have been proposed for sample preparation for CLEM methods. This review focuses on a method that combines light microscopic time-lapse observation or live imaging prior to EM to study cellular dynamics, which is referred to as video CLEM, live CLEM or time-resolved CLEM. Of course, there are many options and technical barriers for each method. This review addresses each issue and describes a practical CLEM method to observe the cellular dynamics in 3D using focused ion beam (FIB)-SEM tomography [4].

## Live CLEM solutions using FIB-SEM and other techniques

The key challenges of the CLEM method are relocating the fluorescence probes with electron microscopic level ultrastructure. Most recent common CLEM approaches involve fluorescence-labeled targets, which can observe the location of functional molecules in living cells. However, the functional observability is inconsistent with its ultrastructural morphology. For CLEM with fluorescent probes, when samples are being prepared, careful attention must be paid to both the retention of immunoreactivity or fluorescence on the target and the preservation of its morphology to relocate the location of fluorescence under its ultrastructure. This relocation step usually creates technical barriers in CLEM, i.e. identifying a specific location in the 3D space under the electron microscope. The thickness of the specimens for light microscopy is approximately a few micrometers, even in the case of culture cells, and the target molecule is located in such a space. When such a specimen is observed by TEM, the distribution of the target molecule is only 1/100th that of an optical microscope because the thickness of the section for conventional TEM is only tens of nanometers. There are three approaches to relocate the target area (Fig. 1). One is to perform CLEM on the same section as the Takayasu cryo-section [5] or plastic embedded section [6–8], allowing the direct observation of the fluorescence on the section for EM and making the relocation of the fluorescence to the ultrastructure relatively easy. In contrast, it is necessary to configure delicate conditions for sample fixation and embedding and probe characteristics to correlate with the well-preserved ultrastructure. The most common method to correlate the target with the ultrastructure is the observation of serial ultrathin sections under TEM. However, the preparation of complete serial sections of cultured cells requires skilled techniques and is time-consuming, and the distortion of sections sometimes makes it difficult to make a correlation. The last approach is using automated 3D reconstruction methods such as FIB-SEM tomography [4, 9]. Both data acquisition and correlation are relatively easy; however, this method requires specialized equipment.

### Fluorescence labeling for live CLEM using FIB-SEM and other live CLEM workflows

The combination of genetically encoded fluorescent proteins, such as GFP, has provided important advances in the field of CLEM, particularly in the field of live imaging CLEM [10]. The use of fluorescent proteins not only allows us to observe the dynamics of proteins, but also solves a major problem of structure preservation in CLEM. Their fluorescence can be observed under a fluorescence microscope both in the live state and after fixation for EM sample preparation, such as glutaraldehyde chemical fixation [4] or cryo-fixation [11]. Autofluorescence of the specimen is known to increase after glutaraldehyde fixation [12], but it does not interfere with the observation of the fluorescence of tag proteins in the case of cultured cells, because the thickness of the cells is thinner than the tissue sections, and it is considered that the amount of fluorescence from fluorescent proteins and autofluorescence has a higher signal-to-noise ratio. Therefore, cellular dynamics can be continuously observed from the live state to immediately after fixation, and the snapshot of the cellular event can correlate with its ultrastructure. It should be noted that such fluorescence is mostly quenched by following osmium tetroxide treatment, which is essential for sample preparation for FIB-SEM observation. If you try to perform CLEM on sections including array tomography (AT), fluorescence must be maintained after embedding in resin [13]. Careful optimization of fixation and embedding conditions [6] or restoration of fluorescence by alkaline treatment after thin sectioning allowed for CLEM on sections [7]. The use of osmium-resistant florescence protein also provides the possibility of CLEM on section [8]. However, when performing CLEM in volume SEM, there is no opportunity to observe the fluorescence in the resin (Fig. 1).

Another way to relocate the location of the fluorescence is to visualize the site of protein localization under an electron microscope using enzyme histochemistry or photochemistry. APEX2 is a soybean ascorbate peroxidase-derived genetic tag, and the location of the tagged protein can be visualized by enzymatic reaction with 3,3ʹ-diaminobenzidine (DAB) into an insoluble osmiophilic polymer at the site of the tag. Because APEX2 does not have any inherent fluorescence, a molecular approach of fusing GFP gene or GFP-binding peptide gene is used to visualize them under light microscopy and EM [14–16]. Photooxidation has also been developed as a tool for CLEM to visualize fluorescent dyes by converting them into DAB reaction products [17]. Fluorescent proteins, such as miniSog, generate free radicals during illumination and capture DAB to produce fine granular precipitates [18]. DAB reaction products allow the visualization of the location of the target under an electron microscope, making relocation easier; however, simultaneously, the DAB reaction products mask detailed morphological information. This is a good approach in case the target to be observed is a membrane-wrapped organelle.

**Fig. 2.**
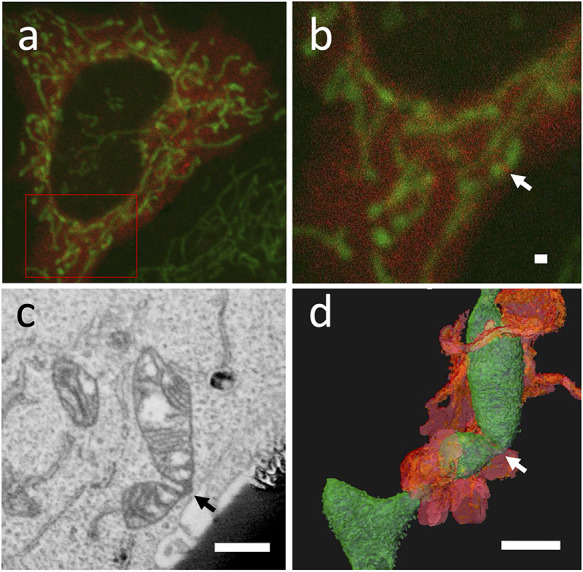
Live 3D CLEM of punctuate of DRP1 in HeLa cell. The cells were labeled with PDHA1-GFP for mitochondria (green) and mCherry-DRP1 (red) (a, b). Punctuate of DRP1 was observed on mitochondria (b arrow). Virtual section obtained from a 3D reconstructed dataset from FIB-SEM tomography data (c), and a 3D view of the dataset from the same direction as the optical microscope (d). The arrow in (c) and (d) correspond to the same locations as the arrow in (b). Green-colored objects denote the mitochondria, and orange-colored objects indicate the surrounding endoplasmic reticulum in d. bar = 1 μm.

If you focus on the morphological preservation of the surrounding area of the target, you can use internal positional markers in addition to the target molecule, which can be observed by both light microscopy and EM to identify the location of the protein on a 10-nm scale based on its relative positional relationship. Fluorescence beads [19] or organelles such as mitochondria [4] can be used as internal fiducial markers. Fig. 2 shows an example of the location of the DRP1 protein around the mitochondria via 3D CLEM using FIB-SEM tomography 3D reconstruction, which allows relocation using the shape of mitochondria between light microscopy and EM (Fig. 2). In any case, it is recommended to select the fluorescent probe to be used depending on the purpose.

### Selection of culture dish for practical live CLEM

A major challenge in the CLEM method is the relocation of intracellular targets in 3D space under EM after live imaging and the following extensive electron microscopic sample preparation procedures. Therefore, a gridded thin-bottom petri dish or gridded coverslip is commonly employed in numerous CLEM studies, which allows easy identification of the target cell. In our method, the Ibidi μ-Dish Grid-500 (Ibidi cat. no. 61166) was used for the culture dish, which is the key to this practical CLEM method.

In most CLEM methods performed at room temperature, the specimens on the coverslip are finally embedded in resin. If a gridded glass-bottom dish is used, the resin-infiltrated culture cells can be removed from the cover glass by heating using a hot plate (105°C) or cooling with liquid nitrogen. However, this step is not always successful in our laboratory, and sometimes the specimen is lost. To increase the success rate, the use of hydrogen fluoride has been proposed to remove glass coverslips from flat-embedded cells [20, 21]; however, hydrogen fluoride is not a practical option because of the hazards involved.

The polymer-bottom gridded dish, i.e. Ibidi μ-Dish Grid-500, can be cut and removed from the specimen directly with a diamond knife, or other precisely controlled trimming tools [22]. Furthermore, in our method, the polymer-bottom film was removed from the sample resin by dissolving it in a solvent for a more practical method. Ibidi μ-Dish is resistant to ethanol and acetone, but not to QY-1 and toluene. Therefore, the cell on the dish can be embedded in resin using a conventional protocol. After polymerization by heating (65°C, 48 h), the 180-μm thick polymer-bottom film was removed by scrubbing with a cotton swab dipped in toluene (Fig. 4a-d). Here, the specimen was dried in an oven (60°C, overnight).

**Fig. 3.**
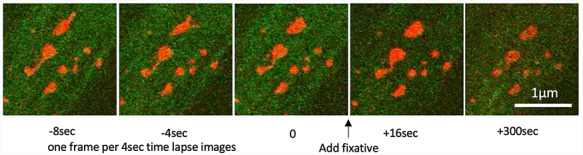
Time-lapse confocal images of mouse embryonic fibroblast (MEF). Mitochondria were genetically labeled with Su9-RFP (red). The cells were fixed immediately after the acquisition of image 0. Mitochondria were completely immobilized by administering the fixative (half Karnovsky solution).

**Fig. 4.**
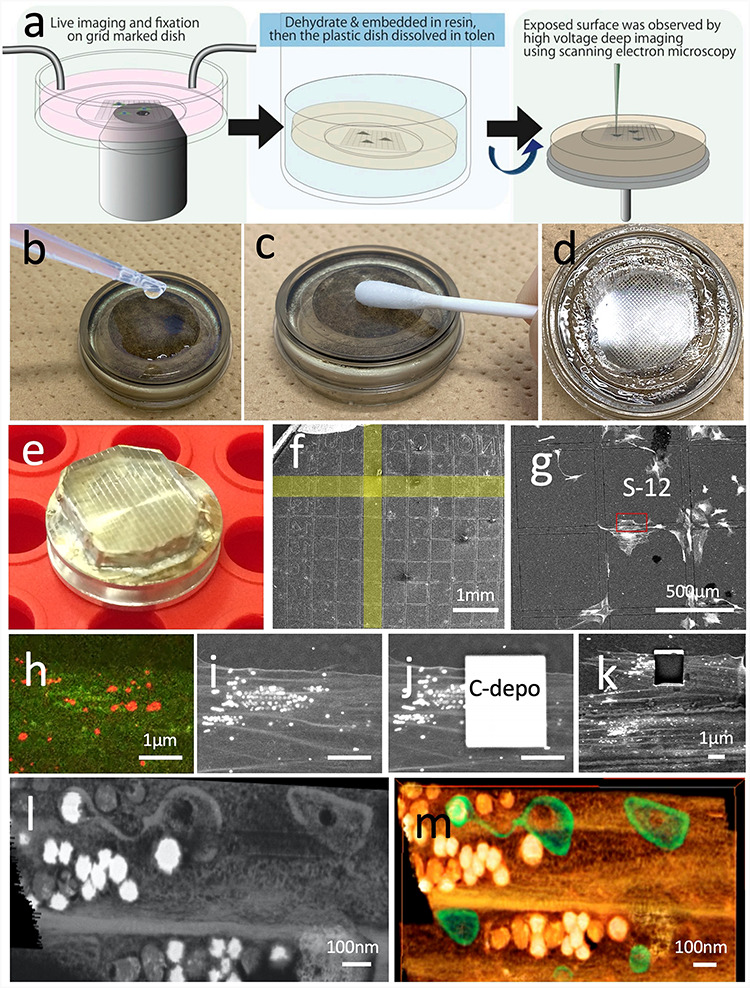
Procedures of 3D CLEM using FIB-SEM tomography. (a) shows the outline of sample preparation (modified from ref. 4 / CC BY 4.0). After embedding the cells in the resin, the bottom of the petri dish was immersed in toluene (b) and swabbed to remove the bottom substrate (c) and expose the resin surface(d). A resin disk was mounted on a sample stub (e) and observed by SEM (f). The target site was easily identified using an imprint of the μ-Dish grid. A high acceleration voltage SEM image (15 kV) determined the cells to be the same as those observed under the optical microscope shown in Fig. 3. Magnified optical microscope images and SEM images of the same area (h, i). Carbon deposition was overlaid on the target area for reconstruction using the gas injection system (j), and the same area after serial images was obtained (k). Virtual slice (l) and volume rendering image (m) of the 3D reconstruction dataset viewed from the same direction as the optical microscope.

### Time-lapse observation to fixation; time resolution for live CLEM

Cell culture at 20–30% confluency is the most preferable cell density for CLEM methods. High density or overlapping of cells makes it difficult to identify the same cells under an electron microscope. Additionally, confluent cultures make a cell thicker, which reduces the accuracy of the correlation using internal fiducial markers by organelles because the markers overlap and are difficult to recognize independently. Thus, we usually seed 1 × 10^5^ cells on a 35-mm dish, which are used for observation on the next day. The dish is held tightly in place with clamps to prevent displacement by liquid exchange or addition of fixing solution during the time-lapse observation.

The point to be considered in time-lapse imaging or live imaging is the same as that of general optical time-lapse observations. It is preferable to reduce the photon dosage during imaging to reduce the effect of phototoxicity on the observation. Confocal laser scanning microscopy should be performed by extending the imaging interval or reducing the intensity of the beam to the minimum requirement. Alternatively, a multiphoton microscope can be employed to reduce the interaction volume. In any case, pre-experiments should be performed to confirm that there are no adverse effects from the irradiation dose. Note that even after fixation, in the acquisition of high-resolution images for correlation after fixation, the morphology may be affected if the beam is too strong.

The fixation process is an important factor in determining the time resolution of this experimental system. Most of the reported live CLEM studies use chemical fixation, while some researchers have used high-pressure freezing (HPF) fixation. These studies have mentioned about membrane trafficking and cell division processes, which could be discussed at a time resolution of seconds to minutes [10, 23]. A method for live CLEM using HPF has been reported to change from last imaging to freezing in a few seconds, although a special sample transfer system is required to proceed from fluorescence microscopy to freezing [24]. In the case of HPF, the time resolution is apparently superior to that of chemical fixation in terms of the morphological conservation because the fixation time is in the order of milliseconds, although several seconds are required from the last image to fixation. Chemical fixation by exposing a jet of fixative to the culture dish may take a few seconds. Here, we have presented a practical example. Fig. 3a shows the time-lapse images obtained every 4 s and the observed mitochondrial morphological transformation. The observation was continued after the addition of fixative (Fig. 3). A quick transformation of the mitochondrial shape was observed within the interval, but the transformation was completely immobilized after fixation and the shape did not change from the last frame of the live image. This means that the estimated fixation period (i.e. time resolution for CLEM) was at least 4 s or less. At this time, fixation was performed by adding a jet to the cell with the same amount of half Karnovsky fixative solution (2% paraformaldehyde, 2.5% glutaraldehyde, 2 mM CaCl_2_ in 0.1 M cacodylate buffer (pH 7.3)) to the culture medium at room temperature (around 25°C) and further fixed for 15 min. We believe that a time resolution in the order of seconds is sufficient for observing mitochondrial dynamics, but if a faster time resolution is necessary, we have to develop a new device such as live observable plunge freezing CLEM.

### Sample preparation for EM

For FIB-SEM tomography, a fixed cell was prepared for the EM sample using a reduced osmium–thiocarbohydrazide–osmium method and en bloc staining of uranyl acetate and Walton’s lead aspartate solution proposed by Deerinck *et al*. [25] to enhance the material contrast of the plasma membrane prior to sectioning. If the EM observation is performed by TEM or AT [26] using SEM, reduced osmium–thiocarbohydrazide–osmium method and en bloc staining are not required. Then, the cells are dehydrated in an ascending ethanol series; they infiltrate the epoxy resin and are cured at 65°C for 2 days. There are two methods to embed the resin. One is a conventional method in which the specimen is embedded at the bottom of the resin block, and it is necessary to remove the dish substrate or film from the resin stub prior to the EM examination. As mentioned above, we used μ-Dish (Ibidi), and all procedures could be performed on the μ-Dish, which can be removed by melting with toluene. The specimen was mounted upside down when performing FIB-SEM and reconstructed from the bottom side. Another embedding method is the thin-layer plastification method, which is proposed to be cured into a film with as little resin as possible so that it can be cut by FIB directly from above the cells [27]. Both methods can reconstruct a specific site for CLEM; however, the former makes it easier to identify if substrate removal is assured.

### 3D reconstruction for CLEM

3D reconstruction at the cellular level for correlation is achievable by both serial section TEM and volume SEM methods. Volume SEM methods are based on an observation of serial sections, which drastically reduce the technical difficulty for reconstruction because they do not required ultrathin sections on fragile grids. In addition, AT and serial block face (SBF)-SEM, also known as the 3View system and FIB-SEM tomography, can be used for 3D CLEM observation. In particular, the FIB-SEM tomography method is convenient for live CLEM in cellular level because it can analyze the 3D architecture of organelles with a spatial resolution of ∼10 nm even in the depth direction, which is suitable for understanding the spatial architecture of biological microenvironments. Additionally, the FIB-SEM machinery can be reconstructed any area that can be observed from the surface. This characteristic makes it possible to select specific sites of specific cells for analysis from a huge block surface of the resin-embedded cell culture, and it is extremely easy to identify the locations observed under an optical microscope (Fig. 4e-i). To observe the resin-embedded cell, SEM observations were performed at a high acceleration voltage of 15 kV, allowing the clear visualization of the shape of the cell attached to the bottom of the culture dish through the embedded resin.

3D reconstruction was performed by repeated cycles of milling on the nanometer scale with an FIB and SEM observation of the newly exposed sample surface. The FIB was irradiated at a perpendicular angle to a smooth specimen to obtain a smooth cross-section that can be observed by SEM to obtain a tissue image [28]. Because the SEM images are obtained from the cross-section of the sample, the angle of observation is 90° different from that of the optical microscope; thus, the correlation after reconstruction must be carefully considered. After determining the location to be reconstructed, a protective layer is formed on the specimen surface with gas injection to allow accurate FIB milling and SEM observation, and then, a cycle of milling and observations is started to obtain a series of sections (Fig. 4j and k). Another advantage of the FIB for CLEM is that, unlike physical cutting with a knife, it can analyze specimens that contain very hard deposits, for example, cells surrounded with silicate glass, or calcium crystal. The milling rate for resin-embedded specimens is ∼10 times higher than that of silicon, and cell embedded resin, for example, can be cut in a few seconds per section.

The maximum size of the reconstruction volume using the gallium ion source FIB is approximately a cube of 100 μm on each side, because the maximum cutting depth is limited to 100 μm from the surface of the specimen. However, when performing CLEM of cell organelles, only a limited target region of the cell should be analyzed, and attempts must be made to increase the spatial resolution as much as possible, for example, a cube of 10 μm on each side with a voxel size of ∼5 × 5 × 10 nm. After aligning thousands of cross-sections, a virtual slice was created using a computer software along the same direction as the optical microscope, and the same mitochondria were observed (Fig. 4l and m). In contrast, the disadvantage of FIB-SEM tomography is that it is difficult to analyze numerous datasets. It takes about a day to perform a single FIB-SEM reconstruction, and the entire equipment is occupied during the process. The following analysis, such as segmentation, takes much more time. The advantage of CLEM, however, is that it can capture a representative example of the phenomena with an optical microscope under an electron microscope, thus providing definitive findings without multiple experiments as when observing each method individually.

### Visualization and correlation

Prior to visualization, the series of images by SEM should be aligned precisely. After alignment, most cases require a segmentation process that extracts the surrounding structures around the target from the volume data. There are many commercial software and open source or GUN public license software (TurboReg, TrackEM2 as ImageJ plugins, Microscopy Image Browser, etc.) for alignment and segmentation. For merging the data obtained from light microscopy and EM, software that can handle anisotropic 3D data such as Amira (Thermo Fisher Scientific) or Dragonfly (Object Research Systems) are preferred. The voxel sizes of both datasets are usually different and not isotropic, and the axial orientation is different between the optical microscope and FIB-SEM volume data. Although merging these data usually requires a complicated process, software that can handle anisotropic data can align them relatively easily by setting the voxel size to match the recorded values. Once the position and direction are aligned approximately, it is relatively easy to correlate with the positional markers by adjusting the data with the positional markers. However, sometimes they may not overlap perfectly because of shrinkage or distortion during EM sample preparation and electrical and geomaterial distortion during imaging. If these are linear deformations, precise correlations can be possible with precise adjustments based on a number of internal positional markers without direct labeling, such as the DAB reaction product.

As mentioned above, there is no technical difficulty in this live 3D CLEM, although an FIB-SEM device is necessary. Thus, no special equipment is required for sample preparation, except for the process of acquiring 3D data with FIB equipment. Moreover, note that, while CLEM can be performed on samples other than cultured cells if the target can be identified by SEM from the bottom of the dish, relocating targets deep in the tissue is challenging and a time-consuming process [29].

## Does mitochondrial uncoupling cause fragmentation or self-fusion? Application of live 3D CLEM analysis for mitochondrial dynamics

Mitochondria are known to be highly dynamic organelles, as described above, that undergo frequent repeat fission and fusion or branching within the cell. The molecular mechanisms of fission and fusion, which induce morphological changes in mitochondria, have been extensively studied, and it has been suggested that reactive oxygen species (ROS) are associated with these morphological changes [30]. A high fission rate induces mitochondria from tubular shape to granular form, which is known as mitochondrial fragmentation. A useful experimental model for mitochondrial fragmentation involves the administration of uncoupler, which induces the globular shape of mitochondria within a few minutes; however, there are some conflicts in its interpretation among EM studies. Briefly, mitochondrial fragmentation is generally accepted as a typical reaction of cells to ROS stresses by differential modulation of mitochondrial fission-fusion proteins [31], and the administration of an uncoupler, such as carbonyl cyanide m-chlorophenylhydrazone (CCCP) or carbonyl cyanide-p-trifluoromethoxyphenylhydrazone, increases proton permeability of the mitochondrial inner membrane and decreases the membrane potential (ΔΨm), which induces ROS stress in the cell [32]. Several light microscopy-based studies have suggested that uncoupling of mitochondria facilitates mitochondrial fission and induces fragmentation [33, 34]. However, such CCCP-administered globular mitochondria frequently exhibit weak fluorescence in their center, and the EM studies have reported that the mitochondria show a ring shape or donut-like shape [35]. Ring-shaped mitochondria have also been reported to be formed via self-fusion of tubular mitochondria because of the loss of ΔΨm [36]. However, it is unclear whether the decrease in mitochondrial membrane potential facilitates mitochondrial fission, fusion, or another mechanism. To address this problem, we performed a live 3D CLEM.

**Fig. 5.**
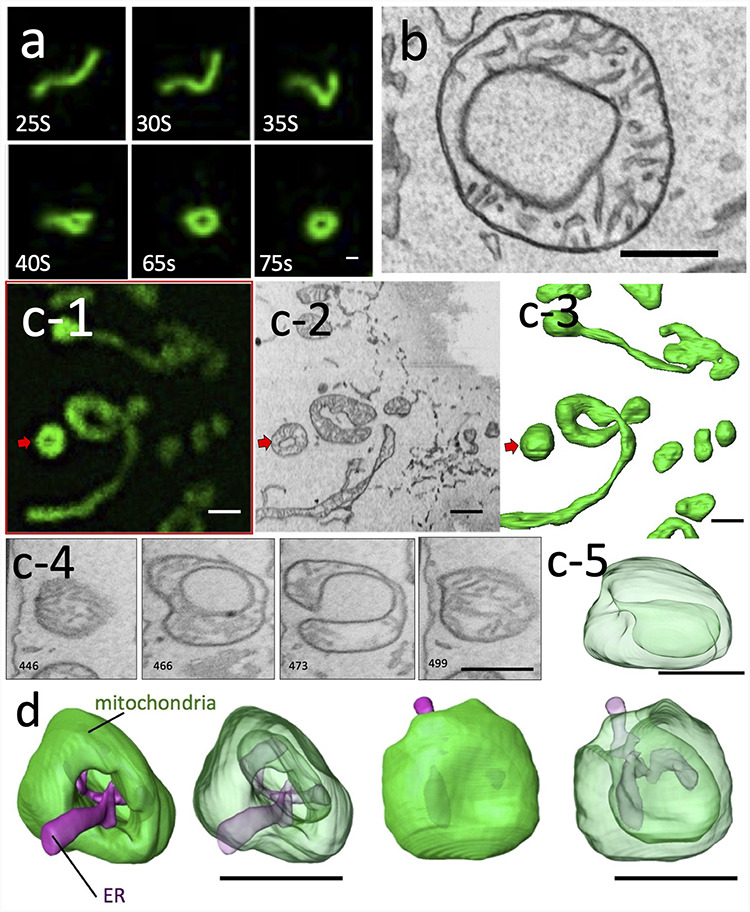
Mitochondrial transformation after 10 μM CCCP administration in MEFs and HeLa cells (Figure adapted from Figs. in ref. no. 4 / CC BY 4.0). Time-lapse images obtained by confocal microscopy (a). The number shown in figures denotes the number of seconds after administration. TEM images of mitochondria in HeLa cells 10 min after treatment with CCCP (b). Live 3D CLEM observation of MEFs after 10 min of CCCP treatment (c). Confocal microscopy image of the transformed mitochondria after time-lapse imaging and fixation (c-1). Virtual cross-section (c-2) and volume-rendered view (c-3) of the area corresponding to c-1. Virtual cross-sections of different levels of mitochondria, shown by red arrows in c-1 to c-3 (c-4). While section 466 shows a ring shape, section 473 shows that the lumen of the ring is connected to the outside, indicating that this mitochondrion is not a true ring shape in 3D. The transparent volume-rendered image demonstrated that this mitochondrion was not a ring, but rather a vase shape (c-5). Transparent volume-rendered images show that vase-shaped mitochondrion have an endoplasmic reticulum in the lumen of vase connected to the external cytoplasm (d). bar = 1 μm.

As shown in Fig. 5, live imaging of the morphological changes in mitochondria after 10 μmol of CCCP showed that the tubular mitochondria transformed into a spherical shape, without fission, as previously reported. Most of these transformed mitochondria had a central area of weak fluorescence, which is characteristic of the ring mitochondria. Immediately after transformation, the cells were fixed, and the subsequent 3D CLEM revealed that many of these were not truly ring-shaped, but rather exhibited a vase-shaped morphology in which the cytoplasm was recessed into the matrix with a small entrance. The structure resembled a ring in one section; however, we were able to determine its true form and revise our interpretation of it via 3D analysis. Such vase-shaped mitochondria have been previously observed by electron tomography [35]; however, 3D CLEM may have revealed the process of their formation. In contrast, because EM can show all morphological information, it is possible to understand the relationships with other organelles, which could not be expected from optical microscopy alone. The results confirm the relationship between the lumen of the vase-shaped mitochondria and the random incorporation of ERs, lysosomes or another mitochondrion. In other words, we suggested that this morphological change did not occur specifically in relation to some structure, but rather was physically random and sucked into the interior by other organelles occasionally attached to the mitochondrial membrane that folded into the lumen. The vase-shaped mitochondrial structures observed in this study are similar to the shape of a stomatocyte, which is known to be physically stable under the condition of a decreased volume–surface ratio compared with that of the sphere shape. The membrane physics study suggested that the stomatocyte shape can be transform from a sphere through an erythrocyte-like biconcave shape when the volume–surface ratio gradually decreases with constant membrane area [37]. Additionally, the stomatocyte formation is also possible when transform from complex to simple structures with a constant membrane area and volume. Therefore, our results strongly suggest that the reduction in mitochondrial membrane potential due to CCCP treatment might have induced a breakdown in the mechanism of mitochondrial shape maintenance. This leads to a morphological change in the physics of surface tension without fission or fusion processes, thus providing an answer that could not be interpreted by conventional optical microscopy and EM alone.

## Conclusion

CLEM, which allows for the observation of temporospatial-specific sites in both function and morphology, is indispensable for future cell biology. Recent studies have revealed that various biological processes proceed by creating a temporal network of organelles and interacting with each other. The observation techniques for organelle dynamics have advanced significantly in recent years, and the study of their dynamics has become more important. Now, CLEM observation is becoming increasingly valuable for understanding microstructures that cannot be captured by optical microscopy. In particular, the combination of live imaging and 3D CLEM will be the key for the future study of organelle dynamics. However, as discussed above, various technical and instrumental barriers exist, making it insufficient for general purposes and for time resolution. Live 3D CLEM using FIB-SEM introduced in this review can partially overcome these technical challenges and have been applied various biological subjects [38, 39]. It is expected that more practical techniques will be developed and can be adapted for many cell biology challenges. In addition, live CLEM will need to be further improved in terms of time resolution. Mitochondrial-derived vesicles captured for the first time by ultrahigh-speed super-resolution microscopy [40] cannot be analyzed in a temporospatial structure without a CLEM with a correspondingly short time resolution.
